# Second Primary Cancers in a Population-Based Mesothelioma Registry

**DOI:** 10.3390/cancers15061746

**Published:** 2023-03-13

**Authors:** Carolina Mensi, Simona Stella, Barbara Dallari, Sabrina Rugarli, Angela Cecilia Pesatori, Giovanni Luca Ceresoli, Dario Consonni

**Affiliations:** 1 Epidemiology Unit, Fondazione IRCCS Ca’ Granda Ospedale Maggiore Policlinico, 20122 Milan, Italy; 2 Department of Clinical and Community Science, Università degli Studi di Milano, 20122 Milan, Italy; 3 Oncology Unit, ASST Valle Olona, Saronno Hospital, 21047 Saronno, Italy

**Keywords:** pleural mesothelioma, second primary cancer, survival, asbestos, genetic susceptibility, cancer registry

## Abstract

**Simple Summary:**

The occurrence of second primary cancers (SPCs) in patients with pleural mesothelioma (PM) included in the Lombardy Mesothelioma Registry (Italy) was investigated, with the aim of assessing its prognostic implications. The results of our study showed that the presence of an SPC in a patient’s history did not significantly impact survival in the overall PM population; however, patients with non-epithelioid PM had a worse prognosis when an SPC was diagnosed. Further studies, including next-generation sequencing of cancer susceptibility genes on germline DNA, are needed to clarify the role of SPCs as markers of genetic susceptibility in mesothelioma.

**Abstract:**

Background: The presence of a second primary cancer (SPC) in patients with pleural mesothelioma (PM) may impact overall survival and suggest a common mechanism of carcinogenesis or an underlying germline genetic alteration. Methods: We evaluated the occurrence of SPCs within PM cases collected from 2000 to 2018 by the Lombardy Mesothelioma Registry and their prognostic implications. Kaplan–Meier analysis was performed to estimate median survival times, together with univariate and multivariate Cox regression models to estimate hazard ratios (HR) and 95% confidence intervals (CI) of death. Results: The median overall survival (OS) of the entire study population (N = 6646) was 10.9 months (95% CI: 10.4–11.2); patient age and histotype were the strongest prognostic factors. No substantial survival difference was observed by the presence of an SPC (10.5 months in 1000 patients with an SPC vs. 10.9 months in 5646 patients in the non-SPC group, HR 1.03, *p* = 0.40). Shorter OS in the SPC group was only observed in 150 patients with the non-epithelioid subtype (median OS of 5.4 vs. 7.1 months, HR 1.21, *p* = 0.03). Conclusions: The diagnosis of an SPC did not influence the outcome of PM patients in the overall study population but was associated with shorter OS in non-epithelioid cases. Further studies are needed to clarify the role of SPCs as markers of genetic susceptibility in mesothelioma.

## 1. Introduction

Mesothelioma is an aggressive tumor arising from the lining membrane of the serous cavities of the body, including the pleura, peritoneum, pericardium, and vaginal tunic of the testicles; pleural mesothelioma (PM) accounts for nearly 90% of diagnosed patients [1]. Most cases of mesothelioma are due to asbestos exposure [2], although other uncommon etiologic factors have been reported [3]. The evidence of familial cases [4] and the occurrence of mesothelioma in patients with no identifiable history of exposure to asbestos or asbestos-like fibers have suggested the possibility of an underlying genetic susceptibility [5,6]. Namely, several studies have demonstrated that carriers of BRCA1-associated protein 1 (BAP1) germline mutations can develop a variety of tumor types, including mesothelioma, uveal and cutaneous melanoma, and renal cell carcinoma; less frequently, breast cancer, different types of skin carcinomas, and other neoplasms have been observed [7]. Interestingly, mesothelioma in patients carrying BAP1 germline mutations is usually diagnosed earlier in life and is much less aggressive than mesotheliomas in the general population [8].

Second primary cancers (SPCs) or multiple cancers have been proposed as possible markers of genetic or familial clustering [9]; indeed, in a study of next-generation sequencing of 85 cancer susceptibility genes on germline DNA from 198 patients with pleural, peritoneal, and tunica vaginalis mesotheliomas, having a diagnosis of SPC significantly increased the odds of carrying a BAP1 germline mutation [5]. Identifying mesothelioma patients with SPCs may therefore have a relevant impact on both prognostic information and familial counseling in cases of an underlying germline alteration. Moreover, investigation of SPCs may help generate hypotheses on the carcinogenic mechanisms of mesothelioma [10].

In this study, we investigated the occurrence of SPCs in the Lombardy Mesothelioma Registry (Registro Mesoteliomi Lombardia, RML), a region in northern Italy that has among the highest number of mesothelioma cases in our country. We focused on PM cases, exploring a possible correlation between SPCs and gender, histological mesothelioma subtype, asbestos exposure, and other demographic variables.

## 2. Methods

### 2.1. The Lombardy Mesothelioma Registry

The Lombardy Mesothelioma Registry is a regional operating center of the National Mesothelioma Registry (Registro Nazionale Mesoteliomi, ReNaM). It opened in 2000 and collects all newly diagnosed cases of mesothelioma among Lombardy residents [11]. Although mesothelioma reporting is compulsory by law (277/1991 and 81/2008), for a complete recognition of mesothelioma incidence, an active search is performed by exploiting several databases, including hospital admissions and mortality.

Mesothelioma diagnosis was assessed after examining medical records and classified according to ReNaM guidelines as “definite” (histological diagnosis, possibly with immunohistochemical confirmation and imaging), “probable” (cytology plus imaging), “possible” (positive imaging), or non-mesothelioma. Qualified personnel interviews confirmed mesothelioma cases or their next-of-kin using a standardized questionnaire to collect lifetime occupational and residential history and occupational history of family members. Following ReNaM guidelines, a group of experts classified asbestos exposure as either occupational (certain, probable, and possible) or extra-occupational. Subjects without any evidence of asbestos exposure were considered unexposed [11].

The presence of a second primary cancer (SPC) was retrieved from the medical records at the time of inclusion in the registry database. The registry collected information on cancer type and date of diagnosis when available. SPCs were recorded according to the International Classification of Diseases, 10th version (ICD-10) [12].

### 2.2. Statistical Analysis

Mesothelioma collection was completed through 2021. For the purpose of this study, all cases of PM diagnosed from 2000 to 2018 were extracted to ensure at least 3 years of mortality follow-up. Kaplan–Meier analysis was conducted to evaluate 3-year survival by the presence of one or more SPC, overall and stratified by PM histological subtype.

Univariate and multivariable Cox regression models were fitted to calculate hazard ratios (HRs) and 95% confidence intervals (CIs) for SPCs adjusted for gender, age (<55, 55–64, 65–74, 75+), year of diagnosis (2000–2004, 2005–2009, 2010–2014, and 2015–2018), diagnostic certainty (“definite”, “probable”, and “possible”), histotype (epithelioid, sarcomatoid, or biphasic—the last two grouped as “non-epithelioid”), asbestos exposure (ever, never), and tobacco smoking history (never, former, current). All analyses were performed using Stata 17 (Stata Corp. 2021, College Station, TX, USA) [13].

## 3. Results

A total of 6688 PM patients registered in the RML from 2000 to 2018 were included in the analysis. Of them, 1005 (15.0%) had a recorded SPC; in the remaining 5683 (85.0%), no SPC was reported. The main characteristics of SPC and non-SPC PM cases are listed in Table 1. In both groups, the majority of patients were males, aged ≥ 75 years, had ever been exposed to asbestos, and were never smokers. Most had a definite diagnosis, with epithelioid histological subtype reported in nearly 60% of cases. The only difference we observed between the two groups was an increasing frequency of PM with SPCs over time.

Of the 1005 patients with an SPC, 103 (10.2%), 12 (1.2%), and 2 (0.2%) had 2, 3, and 4 malignancies, respectively, in addition to PM, for a total of 1138 SPCs. Table 2 reports the distribution of SPCs overall and by gender. Prostate cancer (38.0%), colorectal cancer (11.6%), bladder cancer (10.4%), cutaneous basal cell carcinoma (7.1%), and renal cell cancer (5.1%) were the most frequent neoplasms among men. Among women, the most frequent malignancies were breast cancer (42.2%), uterine carcinoma (8.6%), non-melanoma skin cancer (8.1%), colorectal cancer (6.4%), cutaneous melanoma (5.3%), and thyroid cancer (5.3%). The year of SPC diagnosis was available in 915 out of 1005 cases, for a total of 1042 cancers. Almost all SPCs occurred before their PM diagnosis, but 56 (5.5%) were diagnosed concomitantly with PM (in the same year). Of these, the most frequent were prostate cancer (23.2%), colorectal cancer (14.3%), non-melanoma cutaneous cancers (10.7%), and kidney cancer (7.1%). The distribution of SPCs was similar for epithelioid and non-epithelioid PMs (Table 3), except for breast cancer, which was more frequently associated with epithelioid PM (17.1% vs. 9.3% of total SPCs, *p* = 0.02).

Considering cancers for which there is an established correlation with asbestos exposure, there were 33 (2.9%) cases of laryngeal cancer, 18 (1.6%) cases of lung cancer, and 8 (0.7%) cases of ovary cancer. When we focused on tumors potentially associated with BAP1 syndrome [7], we found 84 (7.4%) cases of cutaneous basal cell carcinoma, 49 (4.3%) of renal cell cancer, 46 (4.0%) of melanoma (42 cutaneous melanoma, 4 uveal melanoma), and 11 (1.0%) of hepatocellular carcinoma.

**Figure 1:**
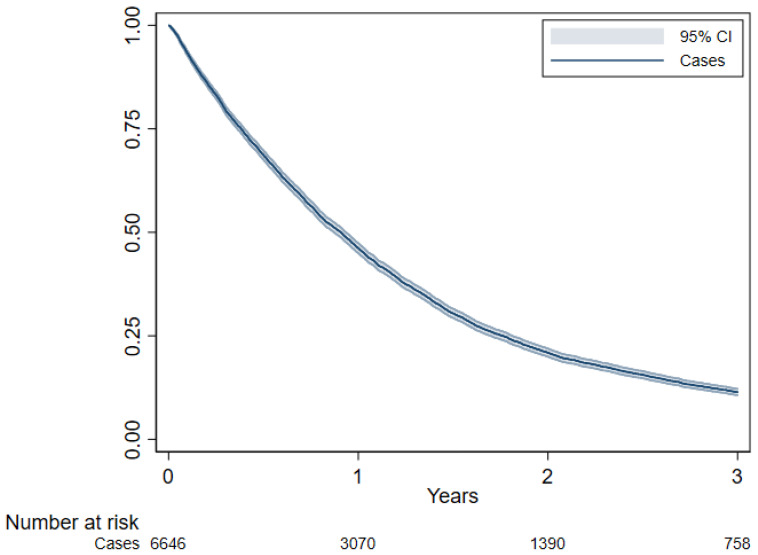
Overall survival estimated using Kaplan–Meier curve, Lombardy, 2000–2018. Follow-up period 2000–2021.

**Figure 2:**
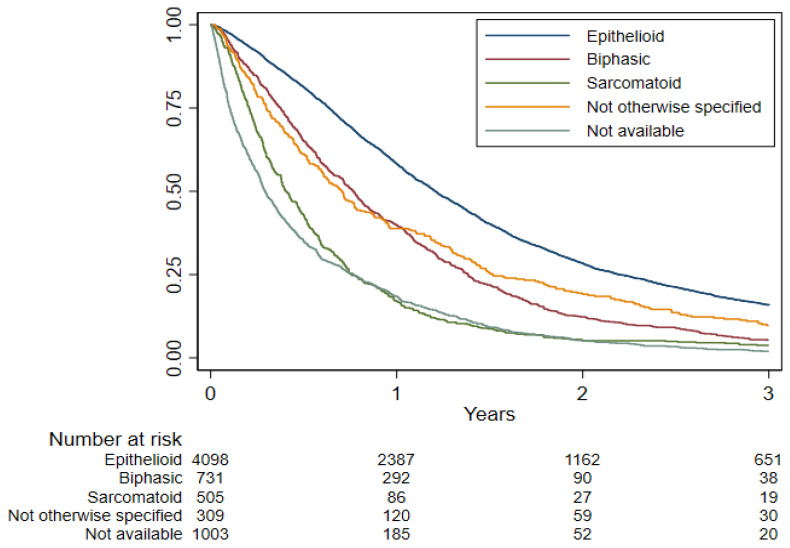
Overall survival by histotype estimated using Kaplan–Meier curve, Lombardy, 2000–2018. Follow-up period 2000–2021.

In survival analyses, 42 cases were excluded because the dates of diagnosis and death were coincident, leaving 6646 subjects in the analysis. The median overall survival (OS) was 10.9 months (95% CI: 10.4–11.2) (Figure 1). Histology, age at diagnosis (particularly age ≥ 75 years), year of diagnosis (with a reduced risk of death from 2005 onward), and level of certainty of diagnosis were the strongest prognostic factors (Table 4). Histologic subtype was confirmed as a paramount prognostic factor, with longer survival observed in patients with epithelioid histotype (Figure 2). Females showed slightly better survival in the adjusted model. Patients with a missing history of asbestos exposure had an increased risk of death.

**Figure 3:**
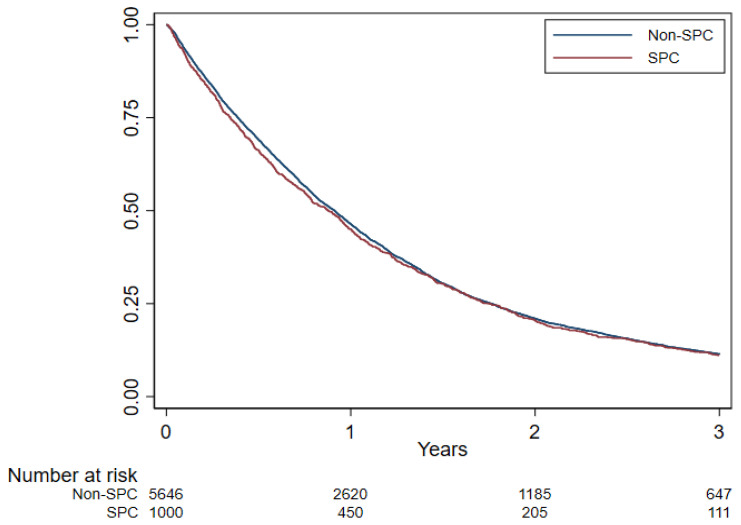
Overall survival by second primary cancer (SPC) estimated using Kaplan–Meier curve, Lombardy, 2000–2018. Follow-up period 2000–2021.

**Figure 4:**
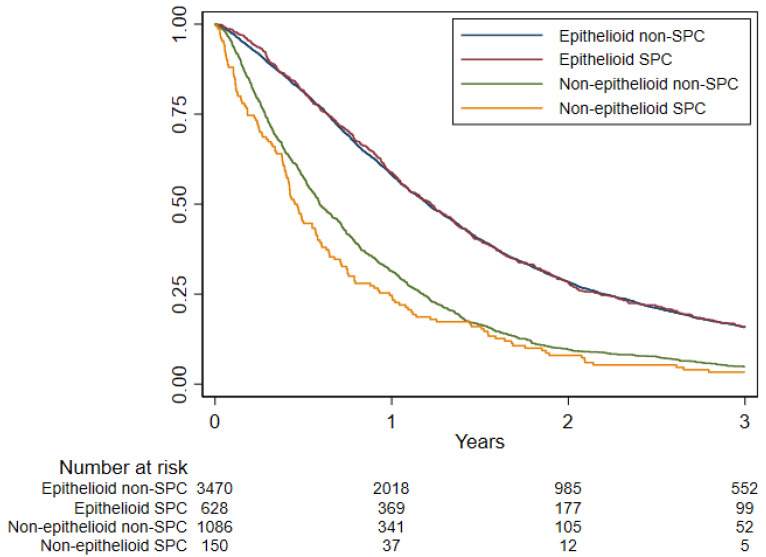
Overall survival among non-SPC and SPC for epithelioid and non-epithelioid histotypes estimated using Kaplan–Meier curve, Lombardy, 2000–2018. Follow-up period 2000–2021.

No substantial OS difference was observed in patients with an SPC (median OS 10.5 months; 3-year OS 11.1%) compared to patients without SPCs (median OS 10.9 months; 3-year OS 11.5%; HR 1.03, *p* = 0.40) (Figure 3; Table 5). Among patients with the non-epithelioid (biphasic or sarcomatoid) phenotype, the OS of patients with SPCs was slightly worse (OS 5.4 months vs. 7.1 months in patients without SPCs; HR 1.21, *p* = 0.03), while no difference between the SPC and no SPC groups was observed in epithelioid patients (Figure 4; Table 5).

## 4. Discussion

The risk of SPC in association with a first malignancy is well known [9,14], particularly with melanoma [15,16,17], skin cancers [18], breast cancer [19], thyroid cancer [20], liver cancer [21], head and neck cancer [22], and mesothelioma [23,24,25,26,27]. In this study, we evaluated the occurrence of SPCs in patients with PM included in a large population-based registry and its possible prognostic role.

Among PM cases collected in the RML, a diagnosis of SPC was reported in 15% of patients. This percentage is far higher than previously reported in other Italian studies on the general cancer population at the national and regional levels [28,29], probably due to different methodologies in retrieving SPC data. In our study, the presence of an SPC was retrieved from the medical records at the time of inclusion in the registry database and also asked about during the interview carried out to collect asbestos exposure history. We observed no gender difference in distribution among the SPC and non-SPC groups; there were approximately twice as many males as females in the analyzed series. As expected, SPCs were more frequent in older patients and in former smokers. Patients with a “possible” PM diagnosis, i.e., without cytologic or histologic confirmation, had a higher rate of SPCs, probably reflecting worse clinical conditions and diagnostic challenges, with difficulty in achieving an adequate bioptic sample in a patient with a previous or concomitant diagnosis of another cancer. Finally, we observed an increase in SPC cases over the years.

Many studies have focused on genetic mutations [5,30] or epigenetic mechanisms [31] of mesothelioma and found common patterns with other cancers. Several findings have suggested a potential role for BAP1 in the carcinogenesis of numerous malignancies [32,33,34,35]. BAP1 is an oncosuppressor protein that acts as a deubiquitinase and is involved in gene expression and chromatin regulation [34,35,36]; it is commonly inactivated in mesotheliomas [7,37,38,39,40]. Germline BAP1 mutations underpin the BAP1 tumor predisposition syndrome (BAP1-TPDS). Some recent studies have suggested that BAP1 mutations may interact with exposure to carcinogens, such as asbestos. This gene–environment interaction is a possible carcinogenic mechanism [34,36,41,42]. Therefore, we evaluated the frequency of asbestos-related and BAP1 syndrome-related malignancies among SPC patients. Interestingly, we found that they comprised nearly a quarter of the entire group of SPCs; among them, the majority were cancers potentially related to BAP1-TPDS.

No difference in survival was found between patients with or without an SPC. However, among PM patients with the non-epithelioid histotype, OS was worse in the SPC subgroup. Our study confirmed the well-established prognostic role of histologic subtype and age at diagnosis in PM [1,3]. Patients with the epithelioid histotype had better survival than non-epithelioid cases; in particular, patients with the sarcomatoid phenotype showed an almost three-fold higher risk of death compared to those with the epithelioid phenotype. Interestingly, patients with unavailable histology had poor survival, likely related to their worse clinical conditions, as discussed above. As already reported [43], patients aged 75 and older showed a worse prognosis, almost a two-fold higher risk of death. Year of diagnosis and degree of diagnostic certainty were other significant prognostic factors in our series. Patients diagnosed before 2005 had an increased risk of death. The improved outcomes of patients diagnosed with PM in more recent years, and particularly after 2005, are at least partially related to the availability of more effective therapies for PM, namely pemetrexed-based chemotherapy [44,45], which represented the cornerstone of PM treatment until the introduction of immunotherapy [46]. The poor outcomes of patients with “possible” PM are likely explained by their worse clinical conditions, which might have precluded an accurate and timely diagnosis of PM. As expected, no difference in survival was observed in association with asbestos exposure [47]. Conversely, patients with missing asbestos or smoking histories had a high risk of death. The majority had no interviews or proxy interviews (with a family member) due to sudden death or serious clinical conditions. Hence, missing information, like missing histotype, is simply an indicator of a severe prognosis.

The main strength of our investigation is the large dataset. The RML is an operative regional center part of a surveillance network, the National Mesothelioma Registry, and has been active since 2000. The registry systematically collects mesothelioma cases throughout Lombardy using standard criteria for the classification of diagnosis and asbestos exposure. Furthermore, RML retrieves information as complete as possible about lifetime occupational and residential history using standard and structured questionnaires [11].

Of course, our study also has some limitations. First, genetic information is not routinely assessed at the time of diagnosis, so we could not perform an analysis of germline mutations related to BAP1 syndrome. Second, the registry retrospectively collects clinical data, including information on SPCs, from medical records, but full clinical data are not always available. Furthermore, in a minority of patients, their inability to sustain a timely interview due to poor clinical conditions or even early death caused a loss of information regarding asbestos exposure, smoking habits, and occupational and residential history. In these cases, close relatives (wife/husband, sons/daughters, siblings) were interviewed.

## 5. Conclusions

In conclusion, in our large population-based series of PM patients, SPCs were common. However, their presence was not associated with a significant change in survival, except for patients with non-epithelioid PM. Consistent percentages of SPCs were potentially asbestos-related and BAP1 syndrome-related; however, genetic analysis is mandatory to demonstrate any germline component in the pathogenesis of these tumors. Non-epithelioid histology and older age were confirmed to be negative prognostic factors in PM.

## Figures and Tables

**Table 1:** Characteristics of pleural mesothelioma patients by presence/absence of a second primary cancer (SPC), Lombardy, 2000–2018.

	Second Primary Cancer		No Second Primary Cancer		*p*-Value
	N	%	N	%	
Total 2000–2018	1005	100	5683	100	
Gender					
Male	682	67.9	3723	65.5	0.15
Female	323	32.1	1960	34.5	
Age (years)					
<55	20	2.0	329	5.8	<0.01
55–64	85	8.5	971	17.1	
65–74	332	33.0	2041	35.9	
75+	568	56.5	2342	41.2	
Diagnosis					
Definite	772	76.8	4603	81.0	<0.01
Probable	75	7.5	405	7.1	
Possible	158	15.7	675	11.9	
Morphology (ICD-O-3 code)					
Not otherwise specified (90503)	42	4.1	275	4.8	<0.01
Sarcomatoid (90513)	61	6.1	448	7.9	
Epithelioid (90523)	630	62.7	3488	61.4	
Biphasic (90533)	90	9.0	649	11.4	
Not available	182	18.1	823	14.5	
Asbestos exposure					
Never	290	28.9	1484	26.1	0.16
Ever	638	63.5	3777	66.5	
Missing	77	7.6	422	7.4	
Tobacco smoking					
Never	432	43.0	2566	45.2	<0.01
Former	438	43.6	2235	39.3	
Current	76	7.5	598	10.5	
Missing	59	5.9	284	5.0	
Year of diagnosis					
2000–2004	115	11.5	1265	22.3	<0.01
2005–2009	208	20.7	1419	25.0	
2010–2014	355	35.3	1672	29.4	
2014–2018	327	32.5	1327	23.3	

Abbreviations: ICD-O-3, International Classification of Diseases for Oncology, 3rd Edition.

**Table 2:** Distribution of second primary cancer (SPC) overall and by gender, Lombardy, 2000–2018.

	Male		Female		All	
	N	%	N	%	N	%
SPC Site/Morphology (ICD-10 Code)	778	100	360	100	1138	100
Pharynx and oral cavity (C02, C09, C10, C11)	9	1.2			9	0.8
Esophagus (C15)	3	0.4	1	0.3	4	0.4
Stomach (C16)	17	2.2	10	2.8	27	2.4
Small intestine (C17)	2	0.3	3	0.8	5	0.4
Colon-rectum (C18–21)	90	11.6	23	6.4	113	9.9
Liver (C22)	8	1.0	3	0.8	11	1.0
Gallbladder (C23)	1	0.1			1	0.1
Pancreas (C25)	4	10.4	2	0.6	6	0.5
Spleen (C26.1)			1	0.3	1	0.1
Larynx (C32)	28	3.6	5	1.4	33	2.9
Lung (C34)	14	1.8	4	1.1	18	1.6
Thymus (C37)			1	0.3	1	0.1
Melanoma (C43)	27	3.5	19	5.3	46	4.0
Other skin cancer (C44, not ICD-O-3 80903)	15	1.9	4	1.1	19	1.7
Skin basal cell carcinoma (C44, ICD-O-3 80903)	55	7.1	29	8.1	84	7.4
Sarcoma (soft tissue and bone) (C46, C49)	10	1.3	2	0.6	12	1.1
Breast (C50)	2	0.3	152	42.2	154	13.5
Uterus, cervix, and endometrium (C53–C55)			31	8.6	31	2.7
Ovary (C56)			8	2.2	8	0.7
Penis (C60)	2	0.3			2	0.2
Prostate (C61)	296	38.0			296	26.0
Testicle (C62)	4	0.5			4	0.4
Kidney (C64)	40	5.1	9	2.5	49	4.3
Bladder (C67, C68)	81	10.4	7	1.9	88	7.7
Lacrimal gland (C69)			1	0.3	1	0.1
Nervous system (C72)	1	0.1			1	0.1
Thyroid (C73)	10	1.3	19	5.3	29	2.5
Adrenal gland (C74)			1	0.3	1	0.1
Parotid gland (C75)	2	0.3	2	0.6	4	0.4
Lymphoma (C81, C82)	35	4.5	17	4.7	52	4.6
Multiple myeloma (C90)	7	0.9	1	0.3	8	0.7
Leukemia (C91–C94)	15	1.9	5	1.4	20	1.8

Abbreviations: ICD-10, International Classification of Diseases, 10th revision; ICD-O-3, International Classification of Diseases for Oncology, 3rd Edition.

**Table 3:** Distribution of second primary cancer (SPC) by histotype, Lombardy, 2000–2018.

	Epithelioid Histotype		Non-Epithelioid Histotype		*p*-Value
	N	%	N	%	
SPC Site/Morphology (ICD-10 Code)	630	100	151	100	
Pharynx and oral cavity (C02, C09, C10, C11)	4	0.6	2	1.3	0.38
Esophagus (C15)	3	0.5	1	0.7	0.77
Stomach (C16)	15	2.4	4	2.7	0.85
Small intestine (C17)	3	0.5			0.40
Colon-rectum (C18–21)	77	12.2	18	11.9	0.92
Liver (C22)	8	1.3	3	2.0	0.50
Pancreas (C25)	4	0.6	1	0.7	0.97
Spleen (C26.1)	1	0.2			0.62
Larynx (C32)	18	2.9	7	4.6	0.27
Lung (C34)	12	1.9	2	1.3	0.63
Thymus (C37)	1	0.2			0.62
Melanoma (C43)	35	5.6	6	4.0	0.43
Other skin cancer (C44, not ICD-O 80903)	9	1.4	2	1.3	0.92
Skin basal cell carcinoma (C44, ICD-O 80903)	48	7.6	14	9.3	0.50
Sarcoma (soft tissue and bone) (C46, C49)	9	1.4	2	1.3	0.92
Breast (C50)	108	17.1	14	9.3	0.02
Uterus, cervix, and endometrium (C53–C55)	20	3.2	1	0.7	0.09
Ovary (C56)	5	0.8	1	0.7	0.87
Penis (C60)	1	0.2			0.62
Prostate (C61)	190	30.2	48	31.8	0.70
Testicle (C62)	3	0.5			0.40
Kidney (C64)	31	4.9	6	4.0	0.62
Bladder (C67, C68)	41	6.5	16	10.6	0.08
Lacrimal gland (C69)	1	0.2			0.62
Nervous system (C72)			1	0.7	0.04
Thyroid (C73)	13	2.1	6	4.0	0.17
Adrenal gland (C74)	1	0.2			0.62
Parotid gland (C75)	4	0.6			0.33
Lymphoma (C81, C82)	39	6.2	7	4.6	0.47
Multiple myeloma (C90)	6	1.0	1	0.7	0.73
Leukemia (C91–C94)	13	2.1	5	3.3	0.36

Abbreviations: ICD-10, International Classification of Diseases, 10th revision; ICD-O-3, International Classification of Diseases for Oncology, 3rd Edition.

**Table 4:** Prognostic factor analysis in the entire study population, Lombardy 2000–2018. Follow-up period 2000–2021.

Variable	N ^a^	Crude HR	95% CI ^b^	Adjusted HR ^c^	95% CI ^b^
Gender					
Male	4375	1	reference	1	reference
Female	2271	1.00	0.95–1.06	0.94	0.89–1.00
Age (years)					
<55	348	1	reference	1	reference
55–64	1052	1.21	1.06–1.39	1.21	1.06–1.39
65–74	2367	1.40	1.23–1.59	1.44	1.27–1.64
75+	2879	2.06	1.82–2.34	1.84	1.62–2.10
Diagnosis					
Definite	5375	1	reference	1	reference
Probable	480	1.77	1.61–1.95	1.16	1.01–1.32
Possible	833	2.72	2.52–2.93	1.39	1.18–1.63
Morphology (ICD-O-3)					
Not otherwise specified (90503)	309	1.46	1.29–1.65	1.29	1.14–1.47
Sarcomatoid (90513)	505	2.71	2.46–2.98	2.66	2.41–2.93
Epithelioid (90523)	4098	1	reference	1	reference
Biphasic (90533)	731	1.64	1.51–1.78	1.68	1.55–1.83
Not available	1003	3.08	2.86–3.30	1.94	1.65–2.27
Asbestos exposure					
No	1768	1	reference	1	reference
Yes	4380	1.02	0.96–1.08	0.99	0.93–1.05
Missing	498	1.66	1.50–1.84	1.32	1.16–1.49
Tobacco smoking					
No	2983	1	reference	1	reference
Yes (former and current)	3325	0.94	0.90–1.00	0.98	0.93–1.04
Missing	338	1.49	1.32–1.67	1.00	0.87–1.15
Year of diagnosis					
2000–2004	1375	1	reference	1	reference
2005–2009	1613	0.85	0.79–0.92	0.87	0.80–0.94
2010–2014	2011	0.88	0.82–0.94	0.85	0.79–0.92
2015–2018	1647	0.89	0.82–0.96	0.81	0.75–0.88
Second primary cancer					
No	5646	1	reference	1	reference
Yes	1000	1.03	0.96–1.11	1.03	0.96–1.11

Abbreviations: ICD-O-3, International Classification of Diseases for Oncology, 3rd Edition. ^a^ Excluding 42 cases with date of diagnosis coincident with date of death. ^b^ CI, confidence interval. ^c^ Hazard ratios (HRs) calculated with multivariable Cox regression models adjusted for age, type of diagnosis, morphology, asbestos exposure, tobacco smoking, year of diagnosis, and second primary cancer (presence/absence).

**Table 5:** Overall survival by second primary cancers (SPC), histotype, and by SPC/histotype jointly, Lombardy 2000–2018. Follow-up period 2000–2021.

	N ^a^	Median Survival (Months)	95% CI ^b^	*p*-Value	Survival Probability (%) 3 Years after Diagnosis
Overall	6646	10.9	10.4–11.2		11.4
Non-SPC	5646	10.9	10.5–11.3	0.40	11.5
SPC	1000	10.5	9.4–11.5		11.1
Morphology (ICD-O-3)					
Not otherwise specified (90503)	309	8.5	7.2–9.4	<0.01	9.7
Sarcomatoid (90513)	505	4.8	4.5–5.6		3.8
Biphasic (90523)	731	9.2	8.5–9.8		5.2
Epithelioid (90533)	4098	14.5	14.1–14.9		15.9
Not available	1003	3.5	3.2–4.0		2.0
Epithelioid histotype					
Non-SPC	3470	14.5	14.0–15.0	0.94	15.9
SPC	628	14.7	13.1–16.0		15.8
Biphasic histotype					
Non-SPC	642	9.3	8.6–10.0	0.62	5.3
SPC	89	7.7	5.6–10.5		5.6
Sarcomatoid histotype					
Non-SPC	444	5.1	4.5–5.8	<0.01	4.3
SPC	61	4.5	2.2–5.1		-
Non-epithelioid histotype					
Non-SPC	1086	7.1	6.7–7.9	0.03	4.8
SPC	150	5.4	4.8–6.8		3.3

Abbreviations: ICD-O-3, International Classification of Diseases for Oncology, 3rd Edition. ^a^ Excluding 42 cases with date of diagnosis coincident with date of death. ^b^ CI, confidence interval.

## Data Availability

The data presented in this study are available on request from the corresponding author.
